# Exchange protein directly activated by cAMP (Epac) protects against airway inflammation and airway remodeling in asthmatic mice

**DOI:** 10.1186/s12931-019-1260-2

**Published:** 2019-12-18

**Authors:** Yi-fei Chen, Ge Huang, Yi-min Wang, Ming Cheng, Fang-fang Zhu, Jin-nan Zhong, Ya-dong Gao

**Affiliations:** 1 Department of Respiratory and Critical Care Medicine, Zhongnan Hospital of Wuhan University, Donghu Road 169, Wuhan, 430071 People’s Republic of China; 2 Department of Intensive Care Unit, Zhongnan Hospital of Wuhan University, Donghu Road 169, Wuhan, 430071 People’s Republic of China; 3 Department of Allergology, Zhongnan Hospital of Wuhan University, Donghu Road 169, Wuhan, 430071 People’s Republic of China

**Keywords:** Exchange protein directly activated by cAMP, Airway inflammation, Airway remodeling, Airway smooth muscle cells, Store-operated Ca^2+^ entry

## Abstract

**Background:**

β_2_ receptor agonists induce airway smooth muscle relaxation by increasing intracellular cAMP production. PKA is the traditional downstream signaling pathway of cAMP. Exchange protein directly activated by cAMP (Epac) was identified as another important signaling molecule of cAMP recently. The role of Epac in asthmatic airway inflammation and airway remodeling is unclear.

**Methods:**

We established OVA-sensitized and -challenged acute and chronic asthma mice models to explore the expression of Epac at first. Then, airway inflammation and airway hyperresponsiveness in acute asthma mice model and airway remodeling in chronic asthma mice model were observed respectively after treatment with Epac-selective cAMP analogue 8-pCPT-2′-O-Me-cAMP (8pCPT) and Epac inhibitor ESI-09. Next, the effects of 8pCPT and ESI-09 on the proliferation and apoptosis of in vitro cultured mouse airway smooth muscle cells (ASMCs) were detected with CCK-8 assays and Annexin-V staining. Lastly, the effects of 8pCPT and ESI-09 on store-operated Ca^2+^ entry (SOCE) of ASMCs were examined by confocal Ca^2+^ fluorescence measurement.

**Results:**

We found that in lung tissues of acute and chronic asthma mice models, both mRNA and protein expression of Epac1 and Epac2, two isoforms of Epac, were lower than that of control mice. In acute asthma mice model, the airway inflammatory cell infiltration, Th2 cytokines secretion and airway hyperresponsiveness were significantly attenuated by 8pCPT and aggravated by ESI-09. In chronic asthma mice model, 8pCPT decreased airway inflammatory cell infiltration and airway remodeling indexes such as collagen deposition and airway smooth muscle cell proliferation, while ESI-09 increased airway inflammation and airway remodeling. In vitro cultured mice ASMCs, 8pCPT dose-dependently inhibited, whereas ESI-09 promoted ASMCs proliferation. Interestingly, 8pCPT promoted the apoptosis of ASMCs, whereas ESI-09 had no effect on ASMCs apoptosis. Lastly, confocal Ca^2+^ fluorescence examination found that 8pCPT could inhibit SOCE in ASMCs at 100 μM, and ESI-09 promoted SOCE of ASMCs at 10 μM and 100 μM. In addition, the promoting effect of ESI-09 on ASMCs proliferation was inhibited by store-operated Ca^2+^ channel blocker, SKF-96365.

**Conclusions:**

Our results suggest that Epac has a protecting effect on asthmatic airway inflammation and airway remodeling, and Epac reduces ASMCs proliferation by inhibiting SOCE in part.

## Background

Allergic asthma is characterized by eosinophilic airway inflammation, airway hyperresponsiveness and airway remodeling [1–3]. Currently, β_2_ adrenergic receptor (β_2_-AR) agonists are widely used as bronchodilators for the pharmacological treatment of asthma, although it is recommended to use in combination with glucocorticoids but not use alone, in order to reduce the risk of β_2_ adrenergic receptor desensitization [4, 5].

β_2_-AR belongs to the G protein-coupled receptor (GPCR) superfamily. Upon activation of β_2_-AR, cAMP is synthesized from ATP by stimulating adenyly cyclase (AC), and then acts as a second messenger to initiate different biological processes [6, 7]. Protein kinase A (PKA) is the traditional signaling pathway of cAMP. The role of PKA in airway smooth muscle relaxation, airway inflammation and airway remodeling has been demonstrated by substantial studies [8–10]. Interestingly, there are evidences suggesting that not all effects of cAMP are mediated by PKA [11–13].

Recently, exchange protein directly activated by cAMP (Epac) has been identified as another important downstream signaling molecule of cAMP [14]. Epac has two isoforms, Epac1 and Epac2, which act as guanine exchange factors (GEF) for the small GTPase effector proteins Rap1 and Rap2 [15, 16]. Epac1 is expressed throughout the body, while Epac2 is expressed mainly in the brain, pituitary gland, adrenal gland and pancreas [17, 18]. Previous studies showed that Epac is involved in airway smooth muscle relaxation [13], proliferation [7], phenotype change [10, 19] and cytokines secretion [7], and also fibroblasts proliferation [20], suggesting a possible role of Epac in airway remodeling.

cAMP plays an essential role in the regulation of immune system, including innate and adaptive immunity [6]. Previous studies demonstrated that cAMP is responsible for the suppressive effects of regulatory T cells (Tregs) on dendritic cells (DCs) and effector T cells [21]. Recent studies demonstrated that Epac is involved in the signaling pathway of cAMP, independently or cooperated with PKA [11, 22, 23]. cAMP inhibited cytokine secretion of DCs, and this effect was partly mediated by Epac [24]. Since Tregs and DCs are important players in asthmatic airway inflammation, these data suggested that Epac activation may suppress airway inflammation in asthma. Interestingly, there are evidences to show that Epac may have promoting effects on inflammation [25, 26]. Epac could increase IL-6 and IL-1β secretion by macrophages, promote IL-8 release from human airway smooth muscle cells and up-regulate inflammatory cytokines production from airway epithelia in a mouse model of asthma [25]. These conflicting data suggested the necessity of elucidating the role of Epac on airway inflammation in asthma.

Airway smooth muscle cells (ASMCs) are major players in airway remodeling. Store-operated Ca^2+^ channels (SOCs) are major Ca^2+^ entry pathway of ASMCs, which are activated upon endoplasmic reticulum/sarcoplasmic reticulum (ER/SR) Ca^2+^ store depletion. Store-operated Ca^2+^ entry (SOCE) is involved in different physiological processes of ASMCs by regulating Ca^2+^ store refilling and Ca^2+^ oscillation [27–30]. We previously showed that β_2_ agonists salmeterol and salbutamol could inhibit the proliferation of ASMCs by reducing SOCE [30]. In the present study, we will also elucidate the effects of Epac on the proliferation and SOCE of ASMCs.

## Materials and methods

### Mice models of acute and chronic asthma

Six- to eight-week-old female BALB/c mice were purchased from and maintained in the Animal Biosafety Levels 3 Laboratory, the Center for Animal Experiments of Wuhan University (Wuhan, China) with environmentally controlled and specific pathogen-free conditions, as we described previously [31, 32]. This study was approved by the Animal Care Committee of Wuhan University (Scientific Ethical Approval No. 2013006).

Acute asthma mice model was established as we previously described [31, 32]. Briefly, on days 0 and 14, mice were sensitized by intraperitoneal injection of 100 μg Ovalbumin (OVA, Grade V; Sigma, St. Louis, MO) emulsified in 2.25 mg of aluminum hydroxide (Sigma, St. Louis, MO) in 200 μl of PBS, and then challenged with 100 μg OVA in 50 μl PBS by intranasal delivery for 3 consecutive days (days 25–27). Different groups of mice were intraperitoneally injected with 100 μl Epac-selective cAMP analog 8-pCPT-2′-O-Me-cAMP (8pCPT; 100 μM; Sigma, St. Louis, MO), Epac inhibitor ESI-09 (100 μM, Sigma, St. Louis, MO) or dimethylsulfoxide (DMSO; Sigma, St. Louis, MO) 0.5 h prior to OVA challenge on days 25–27, respectively. The control (naive) mice were sensitized with OVA and challenged with PBS, and intraperitoneally injected with 100 μl DMSO. Chronic asthma mice model was established as described previously [33]. Briefly, on days 0, 7 and 14, mice were sensitized by intraperitoneal injection of 100 μg OVA emulsified in 2.25 mg of aluminum hydroxide in 200 μl of PBS, and then challenged with 100 μg OVA in 50 μl PBS by intranasal delivery for three times a week for consecutive 6 weeks from day 21. Also, different groups of mice were intraperitoneally injected with 100 μl 8pCPT (100 μM), ESI-09 (100 μM) or DMSO 0.5 h prior to OVA challenge, respectively. And the control (naive) mice were sensitized with OVA and challenged with PBS, and intraperitoneally injected with 100 μl DMSO. Most analyses were conducted after 24 h of the last OVA challenge in acute and chronic asthma mouse model.

### Cell count analysis in BALF

Blood was collected following mice sacrifice after 24 h of the last OVA challenge and the serum was separated by centrifugation. And the sera were used for the analysis of cytokines and IgE levels. Bronchoalveolar lavage fluid (BALF) was collected with three instillations of 0.5 ml PBS containing 0.6 mM ethylene diamine tetraacetic acid (EDTA). The BALF was separated by centrifugation and stored at − 80 °C for later analysis. The pellets cells were re-suspended in 1 ml PBS containing 1% bovine serum albumin (BSA) and total cells were counted. Differential cell counts were assessed by May-Gruenwald Giemsa-stained cytospin slides (Jiancheng, Nanjing, China), as we previously described [31, 32].

### Lung histopathologic examination

The intact left lungs were obtained after bronchoalveolar lavage and fixed in 4% buffered paraformaldehyde for 24 h. The specimens were dehydrated and embedded in paraffin prior to cutting into 4 μm tissue sections. Then the sections were analyzed with hematoxylin and eosin (H&E), periodic acid-Schiff (PAS) or Masson trichrome staining. H&E staining was used to assess peribronchial and perivascular inflammatory cell infiltration. PAS staining was used to evaluate goblet cell hyperplasia. And Masson trichrome staining was used to investigate extracellular collagen deposition. Tissue inflammation was quantified according to a subjective scale of 0–3, as we described previously [31]. The inflammatory degree was scored by two independent observers blinded to the experiment. Briefly, grades of 0 to 3 were given: for no inflammation (grade 0), occasional cuffing with inflammatory cells (grade 1), and when most bronchi or vessels were surrounded by a thin layer (1–5 cells deep: grade 2) and a thick layer (> 5 cells deep: grade 3) of inflammatory cells. An increment of 0.5 was given if the inflammation scores were in between 2 grades, and the total inflammation score was calculated by the addition of both peribronchial and perivascular inflammation scores (*n* = 10 airways from 6 to 8 animals). PAS^+^ areas (A_PAS_^+^), Masson^+^ areas (A_Masson_^+^) and basement membrane perimeter (Pbm) were measured using Image Pro Plus 6.0 software. The degree of goblet cell hyperplasia was represented as A_PAS_^+^ per Pbm (A_PAS_^+^/Pbm). And the degree of collagen deposition was represented as A_Masson_^+^ per Pbm (A_Masson_^+^/Pbm).

### Elisa

The BALF was pooled 24 h after the last OVA challenge and stored at − 20 °C. Levels of IL-4, IL-5, IL-13 and IFN-γ in BALF were quantified using a capture ELISA kit according to the manufacturer’s instructions (eBioscience, San Diego, CA). Serum total and OVA-specific IgE levels were measured with antibodies against mouse total and OVA-specific IgE (Biolegend, San Diego, CA) by ELISA.

### Flow cytometry

After BAL, lung tissue cells were cut into small pieces and enzymatically digested with 1 mg/ml collagenase A and filtered through a 100-μm nylon mesh. After lysis of red blood cells with RBC lysis buffer (Sigma-Adrich), cells were washed with RPMI-1640 medium containing 10% fetal bovine serum (FBS, Gibco, USA), pelleted by centrifugation and re-suspended at a concentration of 1 × 10^6^ cells/ml. After stimulation with PMA and ionomycin, cells were incubated with FITC-labeled anti-mouse CD4 (eBioscience, San Diego, CA) for 30 min at 4 °C in the dark. After the last wash, cells were fixed with 100 μl of Fixation Solution (eBiosience, San Diego, CA) and incubated in the dark at room temperature for 20 min. Cells were then permeabilized and incubated with recommended amount of PE-conjugated anti-IFN-γ, IL-4, IL-13, IL-17A (eBioscience, San Diego, CA) in the dark at room temperature for 20 min. Stained cells were re-suspended and analyzed on flow cytometer (Epics Altra; Beckman). Results are expressed as a percentage of positive cells.

### Airway Hyperreactivity measurement

Airway resistance was measured utilizing the FinePointe RC system (Buxco, Wilmington, NC) 24 h after the final OVA challenge, as previously described [31]. In brief, mice were anesthetized and cannulated of the trachea with a 19-gauge beveled metal catheter. Mice were mechanically ventilated with a rate of 130 bpm and a tidal volume of 0.2 ml, and were challenged with 10 μl PBS followed by cumulative doses of acetyl-β–methacholine (3.125, 6.25, 12.5, 25, 50 mg/ml, Sigma-Adrich) using an ultrasonic nebulizer. Maximum resistance values and minimal compliance during a 3-min period following each challenge were recorded.

### Quantitative PCR (qPCR)

Total RNA was extracted from lung tissue using TRIzol reagent (Invitrogen, Carlsbad, CA, USA) and cDNA was synthesized using ReverTra Ace qPCR RT Master Mix (Toyobo, Tokyo, Japan) according to the manufacturer’s instructions. qPCR was conducted in triplicate using SYBR Premix Ex Taq™ (Takara Bio, Inc., Otsu, Japan). The specific primers were: Epac1 (F) 5′-AGA GAT GCC CGA CTT AGC AA-3′, (R) 5′-TTG GTC TGA GGA GAT ACG-3′; Epac2 (F) 5′-ATA AAA GGC CGT TGG AGC GA-3′, (R) 5′-GCC AGG ACA GCA TAC CAG TT-3′; T-bet (F) 5′-GGT GTC TGG GAA GCT GAG AG-3′, (R) 5′-TGA AGG ACA GGA ATG GGA AC-3′; GATA-3 (F) 5′-AGG GAC ATC CTG CGC GAA CTG T-3′, (R) 5′-GCG GCT TTC AGG CTT CAT GGA G-3′. Glyceraldehyde-3-phosphate dehydrogenase (GAPDH) was used as an internal control with the following primers: (F) 5′-TGT GTC CGT CGT GGA TCT GA-3′, (R) 5′-TTG CTG TTG AAG TCG CAG GAG-3′. The thermal cycling conditions were as follows: denaturation at 95 °C for 1 min, followed by 40 cycles of denaturation at 95 °C for 10 s, annealing at 60 °C for 10 s and extension at 72 °C for 10 s. The data were normalized to the internal reference gene GAPDH). The relative expression of the target gene was calculated using the comparative 2^-ΔΔCt^ method.

### Western blot analysis

Protein was extracted from lung tissue using radioimmunoprecipitation assay (RIPA) lysis buffer (Beyotime Institute of Biotechnology, Haimen, China). Total protein (60 μg) was separated by electrophoresis on SDS-PAGE gels, transferred onto PVDF membranes (Millipore Corp., Billerica, MA, USA), blocked with TBST-containing 5% nonfat dried milk at room temperature for 1 h and probed with rabbit anti-mouse Epac1, Eapc2 or GAPDH (1:200, Santa Cruz Biotechnology, Inc.). The membranes were incubated with primary antibody overnight at 4̊C and then with horseradish peroxidase (HRP)-conjugated anti-rabbit secondary antibody (1:5000; Santa Cruz Biotechnology, Inc.) at room temperature for 1 h. Chemiluminescence images were captured with ECL (Beyotime Institute of Biotechnology) using the Fusion Fx7 image acquisition system (Vilber Lourmat, Marne La Vallée, France). The quantification of the bands was performed by densitometry using Image J software.

### Airway smooth muscle cell preparation

Six-week-old Balb/c mice were obtained from the Center for Animal Experiments of Wuhan University, as mentioned above. Culture of mouse bronchial smooth muscle cells was established as we described previously [34]. Briefly, mice were anesthetized, and the bronchi were dissected from mice under sterile condition. After removing adherent connective tissues and epithelia, the smooth muscles were cut into small pieces. The muscle pieces were then put into 2 ml of PBS containing 1 mg/ml collagenase I (Invitrogen, USA) and 1 mg/ml papain (Invitrogen, USA). After enzymatic digestion at 37 °C for 30 min, the suspension was centrifuged and then washed with Dulbecco’s-Modified Eagle’s medium F12 (DMEM; Gibco, Invitrogen, Carlsbad, CA) containing 10% fetal bovine serum (FBS, Gibco, Invitrogen, Carlsbad, CA). Cells were cultured in DMEM medium supplemented with 10% FBS, 100 mg/ml streptomycin and 100 U/ml penicillin at 37 °C in a humidified atmosphere of 5% CO_2_–95% air. After confluent, cells were passaged with 0.25% trypsin-0.02% EDTA solution (Gibco, Invitrogen, Carlsbad, CA). Cells between 3 and 8 passages were used for experiments. The purity of ASMCs was tested by α-SMA antibody staining and obtained a purity of 95%.

### Cell proliferation assay

The proliferation rates of ASMCs was determined using a cell counting kit-8 (CCK-8; Invitrogen, USA). Cells in each group were inoculated in 96-well plates with 1 × 10^4^ cells per well and three repeated wells for each group. Each group was incubated for 0, 24, 48 and 72 h at 37 °C. Then, 10 μl CCK-8 solution was added to each well, and incubation terminated after 2 h. The optical density (OD) value of each well was measured at 450 nm with a microplate reader (Hanbio Biotechnology Co.). The corresponding OD value represents cell proliferation.

### Cell apoptosis assay

The apoptotic rates of ASMCs were determined with Annexin V-FITC staining. Cells were seeded in 6-well plates (1 × 10^6^ cells per well) for 48 h, and then arrested for 24 h with serum-deprived medium. After incubated with 8pCPT or ESI-09 in 10% FBS-containing DMEM, cells were detached with 0.25% trypsin (Gibco, USA) and washed twice with PBS. Cell apoptosis assay was performed following the manufacturer’s instructions (Annexin V-FITC Apoptosis Detection Kit, eBioscience, USA) by using flow cytometry assay.

### Ca^2+^ fluorescence measurement

Intracellular Ca^2+^ levels were assessed with Fluo3-AM fluorescence on a confocal laser microscope as described previously [30, 35, 36]. Briefly, ASMCs were passaged and seeded on glass coverslips with a density of 5 × 10^3^ cells/ml. After grown in 10% FBS-containing DMEM for 48 h, cells were loaded with Fluo3-AM by incubating with 5 μM Fluo3-AM (Dojindo, Japan) and 0.1% pluronic F-127 in physiological salt solution (PSS: 140 mM, NaCl, 10 mM KCl, 1 mM MgCl_2_, 2 mM CaCl_2_, 10 mM HEPES, 10 mM Glucose, pH 7.2) for 30 min at 37 °C. Cells were then washed two times with Ca^2+^-free PSS (PSS that omitted CaCl_2_ and supplemented with 0.2 mM EGTA) for 5 min and plated in a recording chamber containing 1 ml Ca^2+^-free PSS. 10 μM nifedipine was added in the bath solution to block voltage-gated Ca^2+^ channels. Chemicals and Ca^2+^ were directly delivered to the Ca^2+^-free PSS to reach their final concentration. Intracellular Ca^2+^ fluorescence intensity (FI) was measured at room temperature (22 °C) on a confocal system (Leica TCS SP2 AOBS MP, Wetzlar, Germany) with an Ar-Kr laser and mounted on a Leica microscope (DM IRE2, Wetzlar, Germany). Cells were visualized with a 20 × objective lens. On each coverslip, fluorescences of 15–30 cells were recorded simultaneously (excitation at 488 nm and emission at 530 nm), ROI (region of interest) was defined along the boundary of individual cells. Imaging size was 512 × 512 pixels. The laser intensity and photomultiplier gain were kept constant in all measurements, with the pin hole value of 1 airy.

### Statistics

All results are presented as mean ± SEM. One-way ANOVA was utilized for all analyses except for airway hyperreactivity experiments, in which a two-way ANOVA (with the Bonferroni post-test test) was used. A *P* value less than 0.05 was considered statistic significant.

## Results

### Expression of Epac1 and Epac2 in asthma mice

**Fig. 1:**
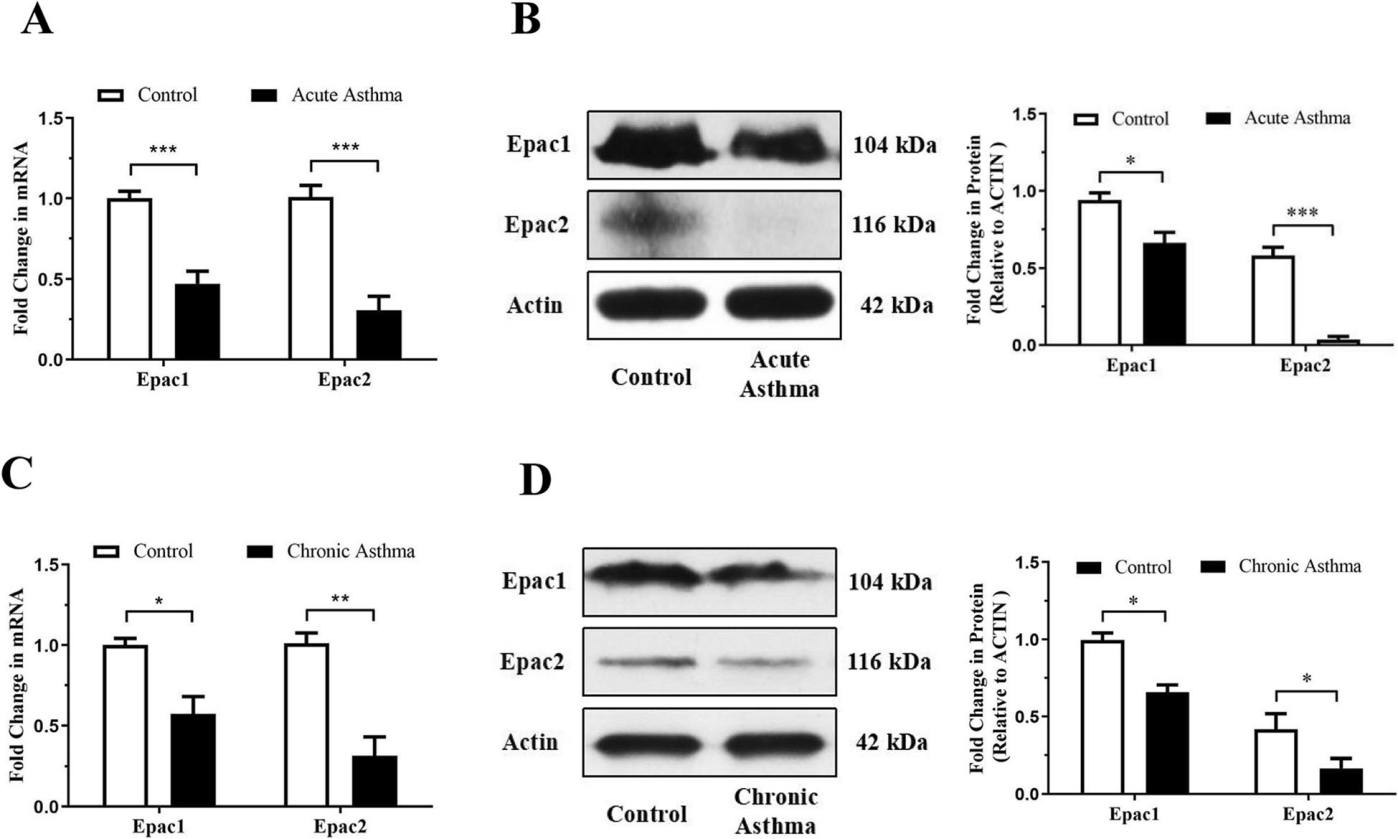
Expression of Epac1 and Epac2 in lung tissues of acute and chronic asthmatic mice. In acute asthma group, female BALB/c mice were sensitized at days 0 and 14 and challenged at days 25–27. Relative expression of Epac1 and Epac2 in lung tissues of acute asthmatic mice was measured by qPCR (**a**) and Western blot (**b**). In chronic asthma group, female BALB/c mice were sensitized at days 0, 7 and 14 and challenged 3 times a week after day 21 for 6 weeks. Relative expression of Epac1 and Epac2 in lung tissues of chronic asthmatic mice was measured by qPCR (**c**) and Western blot (**d**). Actin was used as a loading control. ^*^Control vs Asthma. All data are expressed as mean ± SEM of three independent experiments (*n* = 4–6). ^***^P < 0.001; ^**^P < 0.01; ^*^P < 0.05

To investigate the role of Epac in the regulation of airway inflammation, airway hyperresponsiveness and airway remodeling, we first analyzed the Epac1 and Epac2 expression patterns in lung tissues of acute and chronic asthma mice (Fig. 1). In acute asthma mice, the expression of Epac1 and Epac2 mRNA in the lung was lower than that of control mice, as shown by quantitative PCR (qPCR) (Fig. 1a). A marked decrease in Epac1 and Epac2 expression was also observed at protein level, as shown in Fig. 1b. Similar Epac1 and Epac2 expression patterns were obtained in lung tissues of chronic asthma mice (Fig. 1c, d). These data indicate that a reduction in Epac expression may be associated with airway inflammation, airway hyperresponsiveness and airway remodeling in asthma.

### Effects of Epac regulators on airway inflammation and airway hyperresponsiveness in acute asthmatic mice

Having shown a reduced Epac expression in mice with asthma, we then investigated the effects of Epac regulators on airway inflammation and airway hyperresponsiveness in acute asthma mice model.

#### Effects of 8pCPT and ESI-09 on airway inflammatory cell infiltration and goblet cell hyperplasia

**Fig. 2:**
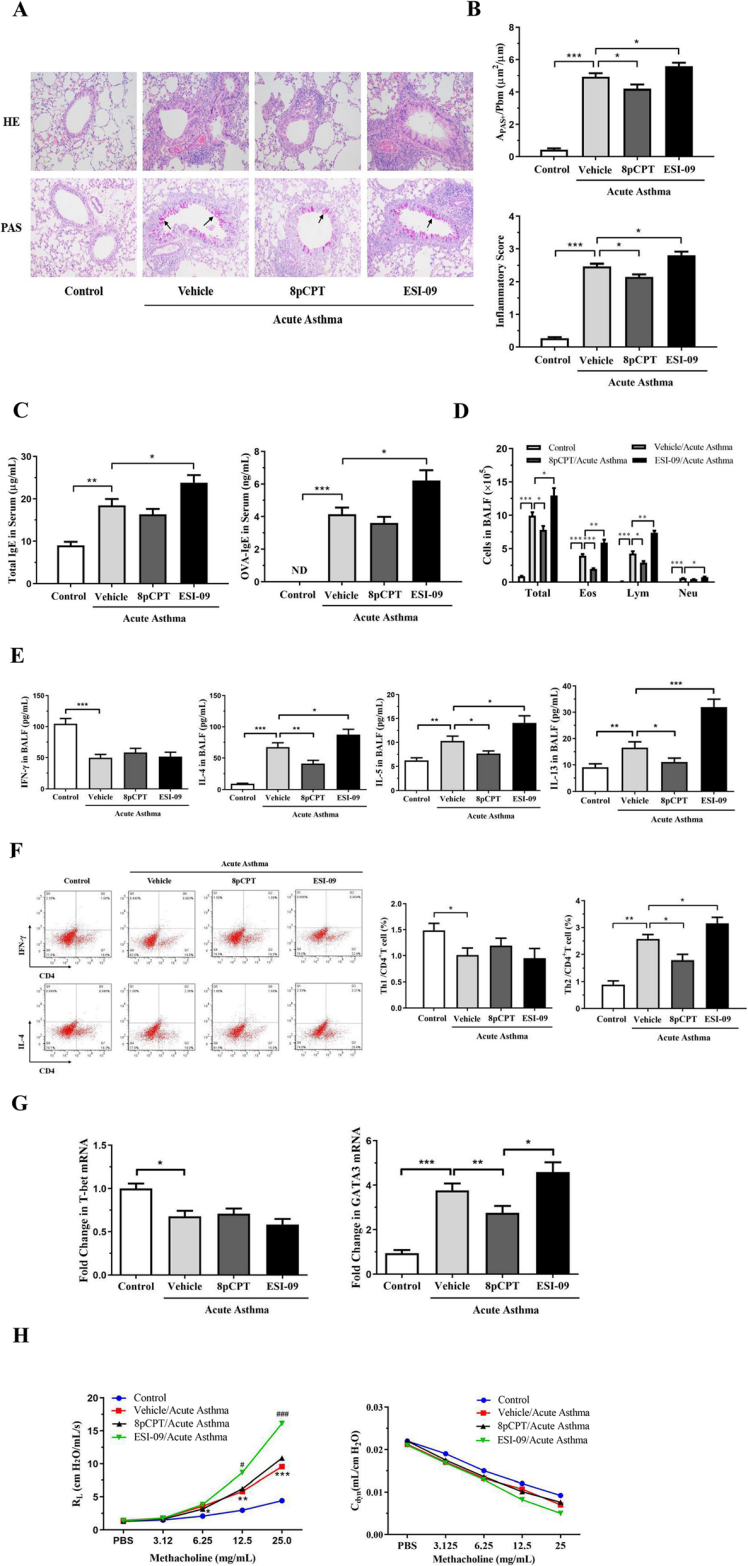
Effects of Epac regulators on airway inflammation and airway hyperresponsiveness in acute asthmatic mice. Female BALB/c mice were sensitized at days 0 and 14 and challenged at days 25–27 in acute asthma group. Mice were received an intratracheal (i.t.). injection of PBS, or an i.t. injection of 25 μg 8-pCPT-2′-O-Me-cAMP (8pCPT), or an intraperitoneal (i.p.). injection of 10 mg/kg ESI-09 30 min before each challenge. Littermates only received allergen challenge as control. Lung tissues were stained with HE and PAS (arrow) (× 200) (**a**). Inflammatory score and PAS-positive areas per unit length were determined (**b**). Total IgE and OVA specific IgE (OVA-IgE) in serum were detected by ELISA (**c**). BALF was analyzed for (**d**) the number of total and different cells (Eos, eosinophils; Lym, lymphocytes; Neu, neutrophils) and (**e**) cytokines (IFN-γ, IL-4, 5 and 13). The frequency of Th1 (CD4^+^IFN-γ^+^) and Th2 (CD4^+^IL-4^+^) cells in lung tissues were detected by FACS (**f**). Lung tissues were measured for transcription factor T-bet and GATA3 mRNA by qPCR (**g**). Airway responsiveness and airway compliance to increased concentration of methacholine (Mch) were measured (**h**). ^*^Control vs Vehicle/Acute Asthma, ^#^Vehicle/Acute Asthma vs ESI-09/Acute Asthma. All data are expressed as mean ± SEM of three independent experiments (*n* = 4–6). ^***^P < 0.001; ^**^P < 0.01; ^*^P < 0.05. ^###^*P* < 0.001; ^#^*P* < 0.05. ND, not detected

In histological analysis, more inflammatory cell infiltration in the peribronchiolar and perivascular zones was observed in OVA-sensitized and -challenged mice (asthma mice) than that in control mice (Fig. 2a). 8pCPT treatment significantly reduced inflammatory cell infiltration in the lung tissues of asthma mice (Fig. 2a). By contrast, mice treated with ESI-09 displayed more inflammatory cell infiltration in the lung tissues (Fig. 2a). Compared with control mice, OVA exposure markedly increased the inflammatory scores of the peribronchial and perivascular region. Reduced inflammatory score in mice treated with 8pCPT and increased inflammatory score in mice treated with ESI-09 were observed (Fig. 2b).

Furthermore, PAS staining was conducted to detect goblet cells. There were no PAS-positive goblet cells in control mice. By contrast, large amount of PAS-positive goblet cells was observed in the airway epithelia of asthma mice, indicating airway mucus hypersecretion. Epac agonist 8pCPT reduced of PAS-positive goblet cells number in airway epithelia. By contrast, Epac inhibitor ESI-09 significantly increased PAS-positive goblet cells number in OVA-exposed mice (Fig. 2a). The PAS-positive areas per unit of length (μm) of the base membrane (A_PAS_^+^/Pbm) was measured quantitatively. A_PAS_^+^/Pbm was higher in OVA-exposed mice than that of control mice, lower in 8pCPT-treated mice, and higher in ESI-09-treated mice respectively than that of OVA-induced asthma mice model (Fig. 2b).

Taken together, these results suggest that Epac activation may have a protecting role in airway inflammatory cell infiltration and goblet cell hyperplasia in OVA-immunized and -challenged acute asthma mice.

#### Effects of 8pCPT and ESI-09 on total and OVA-specific IgE in serum

The levels of total and OVA-specific IgE in serum were measured by ELISA. Only trace amount of total IgE and OVA-specific IgE were detected in control mice. Immunizing and challenging with OVA significantly increased serum level of total IgE and OVA-specific IgE in mice (Fig. 1c). Treatment with 8pCPT in asthma mice slightly decreased total IgE and OVA-specific IgE level but without statistical significance (*P* > 0.05). By contrast, total IgE and OVA-specific IgE levels significantly increased after ESI-09 treatment. These data suggested that Epac inhibition facilitated OVA-specific IgE production and release in the serum.

#### Effects of 8pCPT and ESI-09 on inflammatory cell numbers in BALF

Next, we investigated the effect of Epac regulators on the number of inflammatory cells in BALF. Cellular profiles were assessed by May-Gruenwald-Giemsa staining on cytospin. The numbers of total cells, lymphocytes, neutrophils and eosinophils in BALF were significantly higher in asthma mice than in control cells, consistent with a pattern of airway inflammation (Fig. 1d). 8pCPT treatment significantly reduced the numbers of total cells, lymphocytes and eosinophils. By contrast, ESI-09 significantly increased the numbers of these cells (Fig. 1d). These data suggested that Epac activation may restrain the recruitment of inflammatory cells to lungs and thus attenuate allergic airway inflammation.

#### Effects of 8pCPT and ESI-09 on cytokine levels in BALF

We also checked the effects of 8pCPT and ESI-09 on Th1 and Th2 cytokine levels of BALF by ELISA. As expected, the levels of IL-4, IL-5 and IL-13 were increased after OVA challenge (Fig. 1e). ESI-09 significantly increased, whereas 8pCPT decreased the levels of these cytokines in BALF of asthma mice. The level of Th1 cytokine IFN-γ was slightly lower in asthma mice than that in control mice. In asthma mice, the level of IFN-γ did not change after treatment with 8pCPT and ESI-09 (Fig. 1e).

#### Effects of 8pCPT and ESI-09 on Th1 and Th2 cell numbers in lung tissue

We then checked the percentages of Th1 and Th2 cells in lung tissue with flow cytometry. CD4^+^IFN-γ^+^ T cells and CD4^+^IL-4^+^T cells were regarded as Th1 and Th2 cells respectively. As expected, the percentage of Th1 cells was significantly lower, while the percentage of Th2 cells was significantly higher in asthma mice than that in control mice.

8pCPT and ESI-09 had no effect on percentages of Th1 cells in asthma mice, but they had different effects on Th2 cell frequency of asthma mice. ESI-09 increased, whereas 8pCPT decreased the percentage of Th2 cells in asthma mice (Fig. 1f). These data confirmed the suppressing effect of Epac activation on airway inflammation.

#### Effects of 8pCPT and ESI-09 on T-bet and GATA3 expression in lung tissue

To further clarify the effect of Epac on Th1 and Th2 cells polarization, we investigated mRNA expression of Th1- specific transcription factor T-bet and Th2- specific transcription factor GATA3. The mRNA expression of T-bet was significantly lower in asthma mice than that in control mice (Fig. 1g). 8pCPT and ESI-09 treatment had no effect on T-bet expression. However, GATA3 expression was significantly higher in asthma mice than that in control mice. ESI-09 treatment increased, whereas 8pCPT treatment decreased the expression of GATA3 in asthma mice (Fig. 1g).

#### Effects of 8pCPT and ESI-09 on airway hyperresponsiveness

Finally, we investigated the effects of 8pCPT and ESI-09 treatment on airway hyperresponsiveness of asthma mice by measuring lung resistance (R_L_) to inhaled methacholine. As expected, airway resistance was higher in asthma mice compared to control mice after provoked with methacholine (Fig. 1h). Strikingly, treatment of with ESI-09 significantly increased airway resistance in asthma mice (Fig. 1h). However, treatment with 8pCPT did not change airway resistance in asthma mice significantly. These results implicated that Epac activity may be necessary to inhibit airway hyperresponsiveness.

### Effects of Epac regulators on airway remodeling in chronic asthmatic mice

In addition, we also investigated the effects of Eapc regulators on airway inflammation and airway remodeling in a chronic asthma mice model.

#### Effects of 8pCPT and ESI-09 on airway inflammatory cell infiltration, goblet cell hyperplasia and collagen deposition

**Fig. 3:**
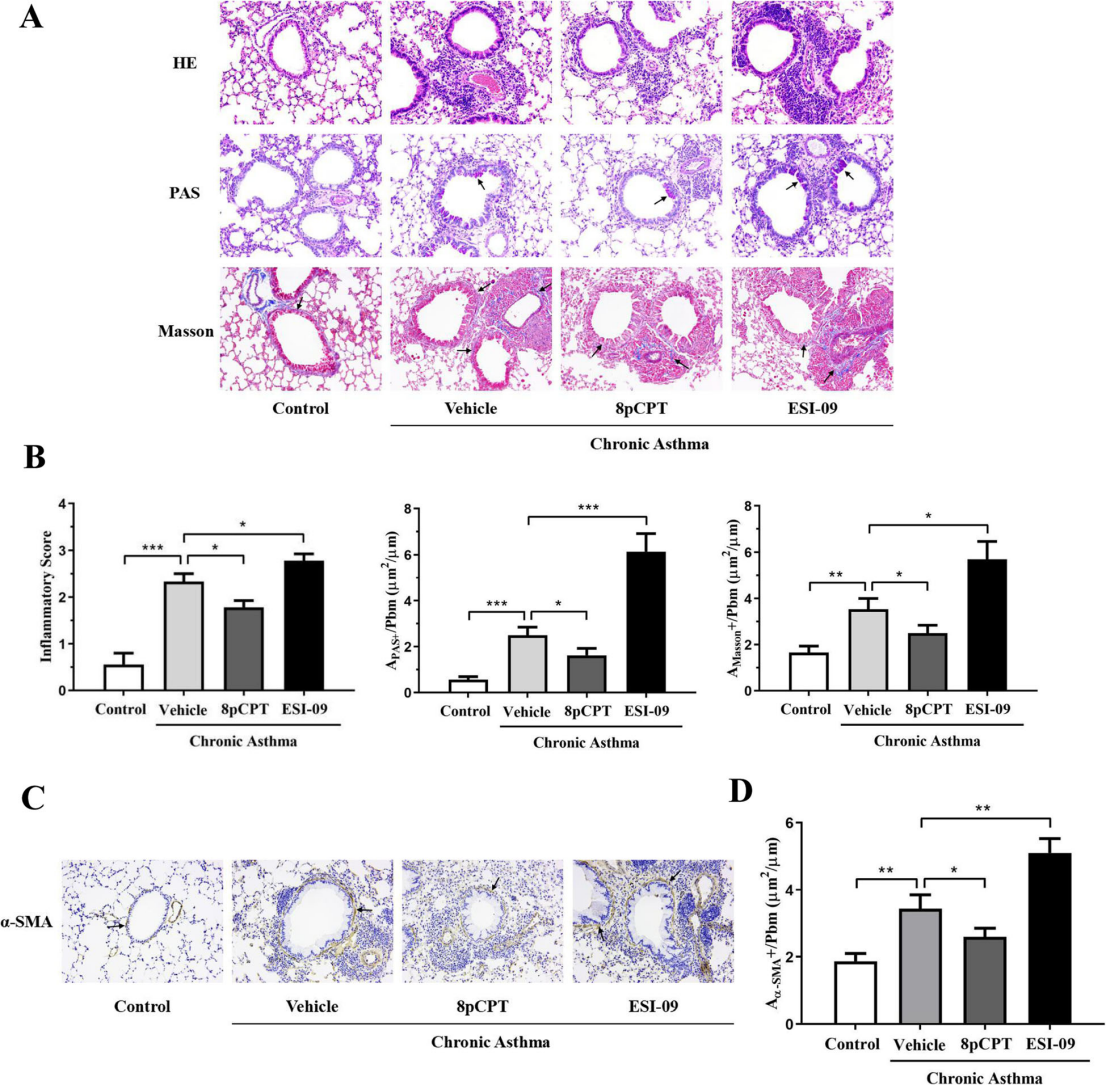
Effects of Epac regulators on airway remodeling in chronic asthmatic mice. Female BALB/c mice were sensitized at days 0, 7 and 14, and challenged 3 times a week for 6 weeks after day 21 in chronic asthma group. Mice were received an intratracheal (i.t.). injection of PBS, or an i.t. injection of 25 μg 8-pCPT-2′-O-Me-cAMP (8pCPT), or an intraperitoneal (i.p.). injection of 10 mg/kg ESI-09 30 min before each challenge. Littermates only received allergen challenge as control. Lung sections were stained with HE, PAS and Masson (arrow) (× 200) (**a**). Inflammatory score, PAS-positive and Masson-positive areas per unit length were determined (**b**). Furthermore, α-SMA, mainly expressed on airway smooth cells, were analysed in lung tissues by immunohistochemistry (arrow) (× 200) (**c**), and α-SMA-positive areas per unit length were determined (**d**). All data are expressed as mean ± SEM of three independent experiments (*n* = 6–8). ^***^P < 0.001; ^**^P < 0.01; ^*^P < 0.05

In chronic asthma mice model, we found more inflammatory cell infiltration in the peribronchiolar and perivascular zones than that in control mice (Fig. 3a). 8pCPT treatment significantly reduced inflammatory cell infiltration in the lung of asthma mice (Fig. 3a). By contrast, treatment with ESI-09 increased inflammatory cell infiltration in the lung of asthma mice (Fig. 3a). Similar results were found with inflammatory scores (Fig. 3b).

Fewer PAS-positive goblet cells were observed in control mice. By contrast, large amount of PAS-positive goblet cells was observed in airway of asthma mice, indicating airway mucus hypersecretion of asthma. 8pCPT reduced, whereas ESI-09 significantly increased the numbers of PAS-positive goblet cells in airway asthma mice (Fig. 3a). Similar results were obtained by quantitative parameter of A_PAS_^+^/Pbm (Fig. 3b).

We then used Masson trichrome staining to determine collagen deposition in airway base membrane. More Masson^+^ area was observed in the peribronchiolar zone in asthma mice than in control mice. 8pCPT reduced, while ESI-09 increased Masson^+^ area in airway of asthma mice (Fig. 3a). The Masson^+^ areas per unit of length (μm) of the base membrane (A_Masson_^+^/Pbm) was used to quantify collagen deposition. Similar results were obtained by A_Masson_+/Pbm (Fig. 3b).

#### Effects of 8pCPT and ESI-09 on airway smooth muscle hyperplasia of asthma mice

ASMCs hyperplasia and hypertrophy was an important feature of airway remodeling in asthma. We then analyzed the expression of α-SMA in lung tissues by immunohistochemistry. More α-SMA-positive cells in the peribronchiolar zone were observed in airway of asthma mice than in control mice. 8pCPT reduced, while ESI-09 increased the number of α-SMA-positive cells in airway of asthma mice (Fig. 3a). The α-SMA-positive areas per unit of length (μm) of the base membrane (A_α-SMA_^+^/Pbm) was used to quantify ASM mass. A_α-SMA_^+^/Pbm was significantly higher in asthma mice than that in control mice. A_α-SMA_^+^/Pbm was lower in asthma mice treated with 8pCPT, and higher in asthma mice treated with ESI-09 (Fig. 3b).

### Effects of Epac regulators on the proliferation and apoptosis of ASMCs

**Fig. 4:**
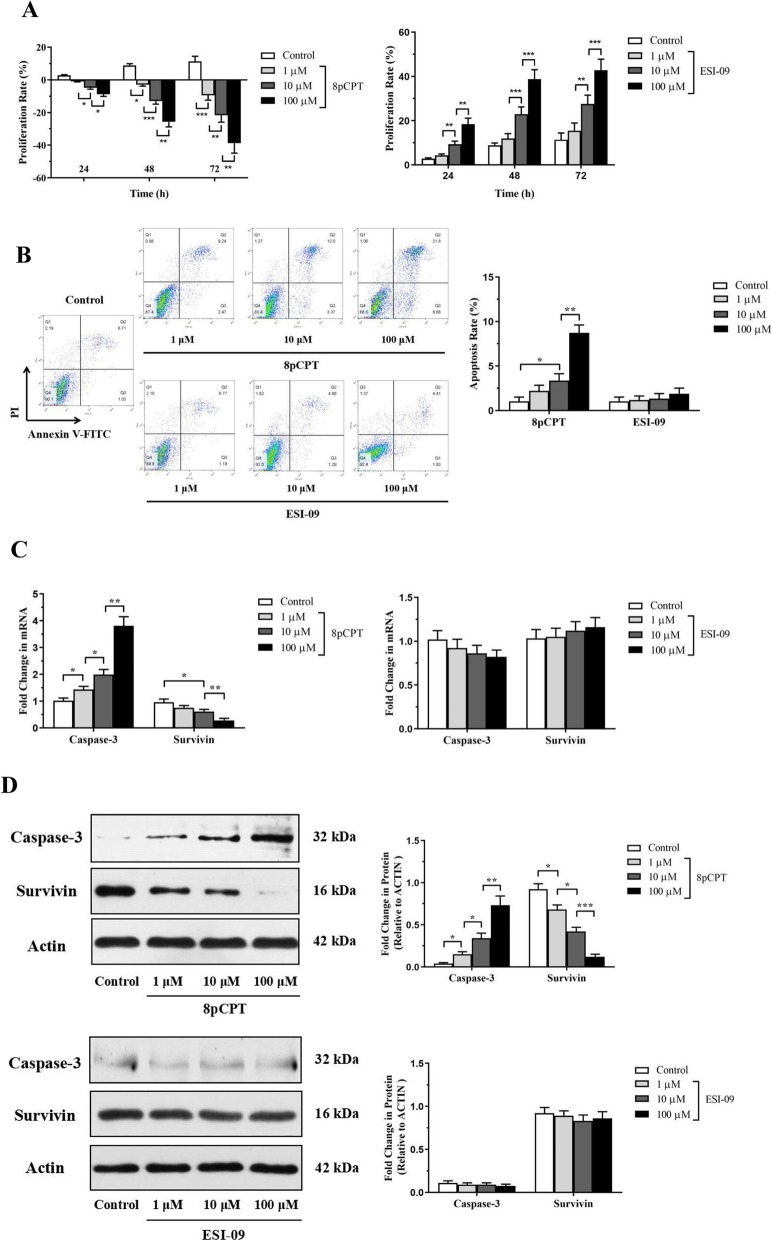
Effects of Epac regulators on the proliferation and apoptosis of ASMCs. Mouse ASMCs were cultured in 10% FBS-containing DMEM and treated with 0, 1, 10, 100 μM 8pCPT or ESI-09 in 96-well plates. The proliferation rate was analysed after 24, 48 and 72 h by CCK-8 assays (**a**). Flow cytometry assays were conducted to detect cells in apoptotic stage at 48 h. Representative dot plots of ASMCs treated with 0, 1, 10, 100 μM 8pCPT or ESI-09 were exhibited (**b**). The apoptotic rates were expressed as the percentages of Annexin V-FITC^+^/PI^−^ cells (**c**). Relative expressions of caspase-3 and survivin were measured by qPCR (**d**) and Western blot (**e**). Actin was used as a loading control. All data are expressed as mean ± SEM of three independent experiments. ^***^P < 0.001; ^**^P < 0.01; ^*^P < 0.05

We next determined the effects of 8pCPT and ESI-09 on the proliferation of in vitro cultured mouse ASMCs. ASMCs cultured in 10% FBS-containing DMEM were treated with 0, 1, 10, 100 μM 8pCPT or ESI-09. 8pCPT dose-dependently inhibited the proliferation of ASMCs after cultured for 24, 48 or 72 h. At 10 and 100 μM, ESI-09 increased the proliferation rate of ASMCs at three different time points (Fig. 4a). The effects of 8pCPT and ESI-09 on ASMCs apoptosis were also investigated with Annexin-V labeling. The apoptotic rates were expressed as the percentages of Annexin V-FITC^+^/PI^−^ cells. Treatment of ASMCs with 8pCPT for 48 h promoted apoptosis of ASMCs at the concentration of 10 and 100 μM, but ESI-09 had no effects on ASMCs apoptosis at any of these concentrations (Fig. 4b).

We then used caspase-3 and survivin expression assay to confirm the effects of Epac regulators on the proliferation and apoptosis of ASMCs. 8pCPT treatment for 48 h increased caspase-3 expression and decreased survivin expression at mRNA level in a dose-dependent manner (Fig. 4c). 8pCPT also increased caspase-3 expression at protein level (Fig. 4d). However, ESI-09 had no obvious effects of on caspase-3 and survivin expression in ASMCs both at mRNA and at protein level (Fig. 4c, d). These data indicate that 8pCPT could inhibit proliferation and promote apoptosis of ASMCs, whereas ESI-09 could promote ASMCs proliferation but had no effects on apoptosis of ASMCs.

### Effects of Epac regulators on SOCE in ASMCs

**Fig. 5:**
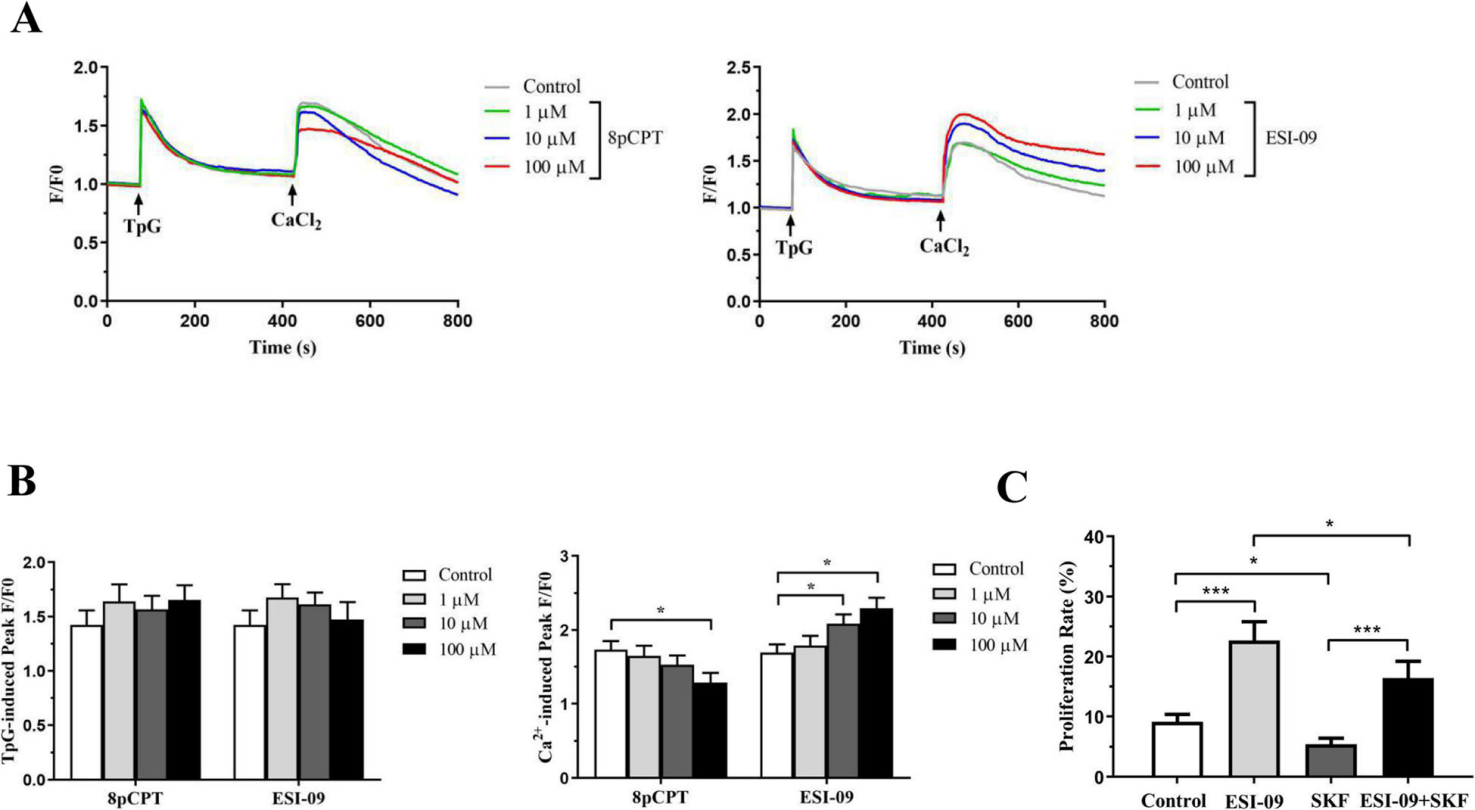
Effects of Epac regulators on SOCE in ASMCs. Intracellular Ca^2+^ concentration was determined with confocal Ca^2+^ measurements, shown are representative time courses of calcium fluorescence changes. Thapsigargin (TpG) was used to induce the depletion of ES/SR Ca^2+^ store, and store-operated Ca^2+^ entry was induced by 2 mM CaCl_2_-containing bath solution. Ca^2+^ fluorescence changes in mouse ASMCs treated with 0, 1, 10, 100 μM 8pCPT or ESI-09 (**a**). Summarized data indicated that both 8pCPT and ESI-09 had no effect on TpG-induced Ca^2+^ release in Ca^2+^-free solution. 8pCPT inhibited store-operated calcium entry (SOCE) only at 100 μM, whereas ESI-09 could promote SOCE at 10 and 100 μM in ASMCs (**b**). 10 μM SKF-96365, the store-operated Ca^2+^ channel blocker, inhibited the promoting effects of ESI-09 on ASMCs proliferation after cultured for 48 h (**c**). SKF: SKF-96365. All data are expressed as mean ± SEM of three independent experiments (*n* = 20–25). ^***^P < 0.001; ^*^P < 0.05

We have previously elucidated the role of store-operated calcium entry (SOCE) in the regulation of multiple physiological processes of ASMCs, especially in cell proliferation and apoptosis. Here we investigated the effects of 8pCPT and ESI-09 on SOCE in ASMCs. Thapsigargin, a non-competitive inhibitor of sarco/endoplasmic reticulum Ca^2+^ ATPase (SERCA), was used to induce Ca^2+^ release from and depletion of ER/SR Ca^2+^ store. 8pCPT or ESI-09 had no effects on thapsigargin (TpG; 0.5 mM; Sigma, St. Louis, MO) - induced Ca^2+^ transient in Ca^2+^- free solution, which indicates Ca^2+^ release. Re-addition of 2 mM Ca^2+^ into bath solution induced store-operated Ca^2+^ entry. ASMCs were treated with 8pCPT or ESI-09 for 30 min at 1, 10 and 100 μM separately prior to Ca^2+^ fluorescence measurements. We found that 8pCPT inhibited SOCE only at 100 μM, whereas ESI-09 promoted SOCE at 10 and 100 μM in ASMCs (Fig. 5a, b). In addition, we demonstrated that the promoting effects of ESI-09 on ASMCs proliferation was inhibited by SOC blocker SKF-96365 (10 μM) after cultured for 48 h (Fig. 5c). These data indicate that SOCE may be involved in the effects of Epac on ASMCs.

## Discussion

In the present study, we demonstrated that Epac activator and inhibitor had opposite effects on airway inflammation and airway remodeling. In lung tissue of asthma mice model, the expression of Epac1 and Epac2 was lower than that in control mice. Epac activator 8pCPT inhibited airway inflammation, while Epac inhibitor ESI-09 promoted airway inflammation in an acute asthma mice model. In chronic asthma mice model, 8pCPT inhibited airway remodeling, but ESI-09 promoted airway remodeling. As to the airway hyperreactivity, 8pCPT had no significant effect on airway resistance induced by methacholine, but ESI-09 dramatically increased airway resistance in acute asthma mice model. In addition, we showed that 8pCPT inhibited, and ESI-09 promoted ASMCs proliferation. 8pCPT promoted apoptosis of ASMCs, but ESI-09 had no effect on apoptosis of ASMCs. Finally, Epac inhibited, while ESI-09 promoted SOCE of ASMCs. SOC channel blocker SKF-96365 inhibited the promoting effects of ESI-09 on ASMCs proliferation, suggesting that Epac may inhibit ASMCs proliferation by acting on SOCE.

As mentioned above, β_2_ agonists are primarily used as bronchodilators in asthma treatment. The non-bronchodilator effects of β_2_ agonists have been reported previously. A study in house dust mite (HDM)-induced allergic asthma mice model showed that long-acting β_2_ agonists (LABA) formoterol effectively reduced airway inflammation and Th2 and Th17 cytokines accumulation [37]. Another study demonstrated that LABA indacaterol, formoterol and salmeterol inhibited IgE-induced release of histamine, leukotriene and prostaglandin D2 release, suggesting additional therapeutic effect of LABA beyond bronchodilator [38].

Lower cAMP concentration in bone marrow-derived dendritic cells (BMDCs) favors a Th2 response and airway inflammation, which is reversed by increasing cAMP concentration and PKA activation [9]. In patients with chronic obstructive pulmonary disease (COPD), the anti-inflammatory effects of cAMP are mediated by PKA and Epac [22]. Study in cigarette smoke-induced COPD models showed that Epac1 primarily inhibits airway remodeling, whereas Epac2 primarily increases inflammatory processes [39]. In the present study, we demonstrated that the expression of Epac1 and Epac2 in lung tissue was down-regulated in asthma mice when compared to control mice. In addition, Epac activator attenuated, whereas Epac inhibitor exaggerated allergic asthma airway inflammation, both in acute and chronic asthma models, suggesting a protecting role of Epac on asthma airway inflammation. Thus, the anti-inflammatory effects of β_2_ agonists are mediated both via PKA and Epac. The distinct role of Eapc1 and Epac2 in asthma airway inflammation should be further investigated.

In chronic asthma mice model, we demonstrated that Epac had a protecting effect on airway remodeling, in that Epac activator 8pCPT inhibited, while Epac inhibitor ESI-09 promoted airway remodeling processes such as goblet cell hyperplasia, collagen deposition and ASMC hyperplasia. This is consistent with the results found in Eapc1^−/−^ and Eapc2^−/−^ mice. The expression of MUC5AC, which is an indicator of mucus production and also an important parameter of airway remodeling, was higher in lung tissues of both Eapc1^−/−^ and Eapc2^−/−^ mice. In Eapc1^−/−^ mice, the expression of fibronectin, collagen I and TGF-β_1_ in lung was higher than that of WT mice, not only in basal level, but also after cigarette smoking exposure [39]. These data implicate an anti-fibrotic effect of Epac, consistent to our results in chronic asthma model. We did not investigate the distinct role of Epac1 and Epac2 in airway remodeling, therefore, it is necessary to establish chronic asthma models on Eapc1^−/−^ and Eapc2^−/−^ mice to confirm our results.

We demonstrated that the Epac activator 8pCPT inhibited, whereas Epac inhibitor ESI-09 promoted mice ASMCs proliferation, suggesting a suppressive effect of Epac on ASMCs proliferation. This is consistent with our previous studies, which showed that short-acing β_2_ agonist salbutamol inhibited the proliferation of human ASMCs and LABA salmeterol inhibited mice ASMCs proliferation [30]. Interestingly, Kassel KM et al. demonstrated that Epac but not PKA is responsible for the inhibiting effect of β_2_ agonists on human ASMCs proliferation [40]. Epac activator 8pCPT inhibited human AMSCs proliferation at a concentration that did not activate PKA (10 μM), this is similar to our results that 8pCPT inhibited mice ASMCs proliferation at 1, 10 and 100 μM. Besides proliferation, we also showed that 8pCPT promoted ASMCs apoptosis, and this effect is not found with ESI-09. Thus, the anti-proliferative effects of 8pCPT, as least in part, should be attributed to its pro-apoptotic effect on ASMCs. The underlying mechanisms of 8pCPT-induced ASMCs apoptosis is not clear and should be further investigated.

β_2_ adrenergic agonists are widely used in the treatment of asthma to relieve bronchospasm. Our previous studies showed that short-acting β_2_ agonist salbutamol inhibited SOCE in ASMCs, and this effect is PKA-dependent, since PKA-selective cAMP analog db-cAMP inhibited SOCE of ASMCs. In addition, we also demonstrated that STIM1- and ORAI1- mediated SOCE is important to ASMCs proliferation. In the present study, we further investigated the effect of Epac signaling on SOCE of ASMCs. Our results showed that Epac activator 8pCPT inhibited SOCE induced by thapsigargin in ASMCs, and this is opposite to the effect of Epac inhibitor, which slightly promoted SOCE of ASMCs. Taken together, these results suggest that Epac and PKA inhibit SOCE cooperatively in ASMCs. The effect of Epac on SOCE was not reported previously in ASMCs, but an inhibiting effect of Epac on SOCE was demonstrated in dystrophic skeletal muscle cells [41] and in rat aorta smooth muscle cells [42].

The mechanisms of Epac on SOCE is still unclear. We found that Epac activator and inhibitor had no effect on thapsigargin-induced Ca^2+^ release, suggesting the effect of Epac on SOCE is not mediated by decrease in ER/SR Ca^2+^ store. This is contrast to a previous study, which showed that PKA and Epac activation induced depletion of Ca^2+^ store in rat aortic smooth muscle cells [42].

## Conclusions

In conclusion, Epac may play a protecting role in asthma airway inflammation and airway remodeling, and the anti-mitogenic effect of Epac on airway smooth muscle cell might be partly mediated by inhibition of SOCE. Our results replenish the underlying mechanisms of β_2_ agonists on airway inflammation and airway remodeling.

## Data Availability

The data generated during the current study are available from the corresponding author on reasonable request.

## Abbreviations

8pCPT: 8-pCPT-2′-O-Me-cAMP
AC: Adenyly cyclase
ASMCs: Airway smooth muscle cells
BALF: Bronchoalveolar lavage fluid
BMDCs: Bone marrow derived dendritic cells
BSA: Bovine serum albumin
CCK-8: Cell counting kit-8
COPD: Chronic obstructive pulmonary disease
DCs: Dendritic cells
DMEM: Dulbecco’s modified eagle medium
DMSO: Dimethylsulfoxide
EDTA: Ethylene diamine tetraacetic acid
Epac: Exchange protein directly activated by cAMP
ER/SR: Endoplasmic reticulum/sarcoplasmic reticulum
FBS: Fetal bovin eserum
FI: Fluorescence intensity
GEF: Guanine exchange factors
GPCR: G protein-coupled receptor
H&E: Hematoxylin and eosin
hASMCs: Human ASMCs
HDM: House dust mite
LABA: Long-acting β_2_ agonists
OD: Optical density
OVA: Ovalbumin
PAS: Periodic acid-Schiff
Pbm: Basement membrane perimeter
PKA: Protein kinase A
PSS: Physiological salt solution
SERCA: Sarco/endoplasmic reticulum Ca^2+^ ATPase
SOCE: Store-operated Ca^2+^ entry
SOCs: Store-operated Ca^2+^ channels
TpG: Thapsigargin
Tregs: Regulatory T cells
β_2_-AR: β_2_ adrenergic receptor
